# Kinase-substrate prediction using an autoregressive model

**DOI:** 10.1016/j.csbj.2025.03.003

**Published:** 2025-03-08

**Authors:** Farzaneh Esmaili, Yongfang Qin, Duolin Wang, Dong Xu

**Affiliations:** Data Science and Informatics Institute, Department of Electrical Engineering and Computer Science and Christopher S. Bond Life Sciences Center, University of Missouri, Columbia, MO 65211, USA

**Keywords:** Phosphorylation, Kinase, Kinase substrate, Autoregressive language model, Zero-shot

## Abstract

Kinase-specific phosphorylation plays a critical role in cellular signaling and various diseases. However, even in model organisms, the substrates of most kinases remain unidentified. Currently, there is no reliable method to predict kinase-substrate relationships. In this study, we introduce an innovative approach leveraging an autoregressive model to predict kinase-substrate pairs. Unlike traditional methods focused on predicting site-specific phosphorylation, our approach addresses kinase-specific protein substrate prediction at the protein level. We redefine this problem as a special type of protein-protein interaction prediction task. Our model integrates protein large language model ESM-2 as the encoder and employs an autoregressive decoder to classify protein-kinase interactions in a binary fashion. We adopted a hard negative strategy, based on kinase embedding distances generated from ESM-2, to compel the model to effectively distinguish positive from negative data. We conducted a top‑k analysis to assess how well our model can prioritize the most likely kinase candidates. Our method is also capable of zero-shot prediction, meaning it can predict substrates for a kinase in case of no known substrates, which cannot be achieved by site-specific prediction methods. Our model’s robust generalization to novel kinase and underrepresented groups showcases its versatility and broad utility. Code and data are available at https://github.com/farz1995/substrate_kinase_prediction.

## Graphical Abstract

**Figure:**
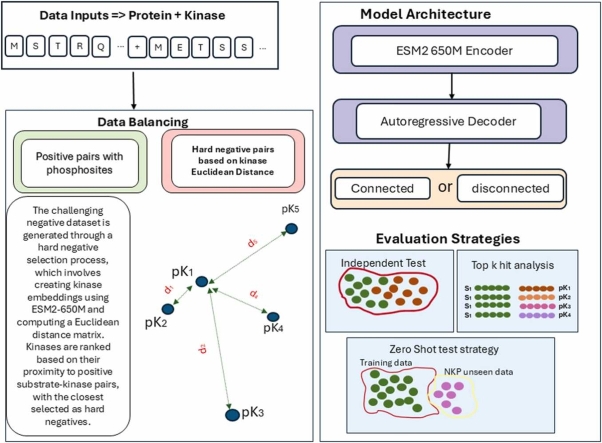

## 1 Introduction

Post-translational modifications (PTMs) are crucial biochemical alterations, playing a vital role in regulating protein functions, stability, and interactions [22]. More than 600 types of PTMs exist, and phosphorylation is the most prevalent and extensively studied [13]. Phosphorylation involves the addition of a phosphate group, typically to serine, threonine, or tyrosine residues within proteins, and is a key regulatory mechanism in cellular signaling pathways [6]. Kinases are specialized enzymes responsible for catalyzing phosphorylation [17]. The specificity of this process, where the right kinase phosphorylates the right substrates at the right time, is essential for maintaining cellular [27]. Dysregulation of kinase activity has been implicated in various diseases, including cancer, diabetes, neurodegenerative, and developmental disorders [3], [9].

While site-specific phosphorylation is critical for understanding cellular signaling, identifying the kinases responsible for phosphorylating specific residues remains a major challenge. Phosphorylation events occur in a hierarchical manner, in which kinase-substrate interactions represent the first step before residue-level modifications take place. A deep understanding of phosphorylation network is crucial for unveiling the mechanisms behind various diseases and identifying suitable targets for medicine development [35], [8]. Traditional computational methods for phosphorylation prediction often focus on site-specific modeling, utilizing sequence motifs or structural context to predict phosphorylation events. However, these models rely on predefined kinase-substrate relationships, which themselves are incomplete and biased toward well-studied kinases. Most of these models rely on using peptides with size of 7–15 amino acids [34], [40], [7], often overlooking the broader interaction between the kinase and its protein substrate. For instance, if the peptide is buried within the core of a protein structure, it becomes inaccessible to kinases and is therefore unlikely to undergo phosphorylation. It is also known that the kinase function is influenced by regions other than the kinase domain, such as regulatory domains, docking motifs, and regions that mediate interactions with substrates or other proteins [25], [31]. Unlike conventional definitions of kinase-specific phosphorylation prediction focusing on phosphorylation sites [13], we redefine this task as a protein-protein interaction (PPI) prediction [11], shifting the focus from individual phosphorylation sites to the broader kinase-substrate interaction landscape. This shift is crucial as it captures the dynamic and functional nature of kinase-substrate interactions, enabling a more comprehensive understanding of cellular signaling processes.

**Fig. 1:**
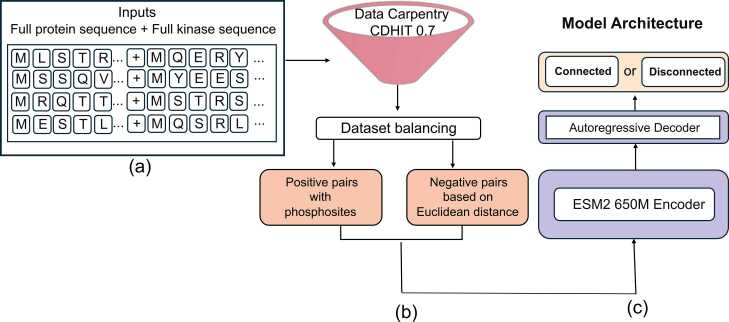
Overview of the model pipeline for kinase-substrate interaction prediction. (a) The model takes full protein sequences and full kinase sequences as input. (b) The dataset is preprocessed using CD-HIT (0.7 threshold) for redundancy reduction, followed by dataset balancing. Positive pairs are selected based on experimentally validated phosphorylation sites, while negative pairs are generated based on Euclidean distance in sequence space. (c) The model architecture consists of an ESM2-650 M encoder that processes input sequences, followed by an autoregressive decoder that predicts whether a given kinase-substrate pair is “connected” (positive pair) or “disconnected” (negative pair).

A potential solution to account for the broader context of kinase-substrate interactions could be the use of autoregressive models, which have shown promise in generating and modeling complex sequences [12], [24], [26], [28], [30], though their application in kinase-specific phosphorylation prediction remains unexplored. Instead of modeling phosphorylation at the peptide or domain level [34], [7], we consider the entire kinase sequence and the full protein substrate sequence to enable more biologically relevant approach to interaction prediction. By integrating our model’s predictions with downstream phosphorylation site prediction frameworks, we can create a more complete phosphorylation network, benefiting both basic biology and translational research [23], [4]. An overview of the study is presented in Fig. 1.

In this paper, we implemented an autoregressive-based model to predict substrate-kinase interaction using either substrate-kinase or substrate-kinase domain input pairs for comparison. We utilized ESM-2 [20]as our encoder, leveraging its training on millions of protein sequences to capture detailed, transferable representations of protein structure and function. For the decoder, we employed an autoregressive approach to predict kinase-specific by determining whether a given protein is a substrate of a specific kinase or not. The model takes protein-kinase pairs as input and outputs a classification indicating whether the protein is "*connected*" *or* "*disconnected*" to the kinase. To improve the model's quality and accuracy, we developed a novel approach for selecting positive and negative samples during training, drawing inspiration from contrastive learning and leveraging Euclidean distances as a guiding metric [39].

We evaluated our model's zero-shot capabilities using two distinct zero-shot datasets (random split dataset and frequency-based stratified dataset), carefully retraining the model to thoroughly assess its generalization ability. To further validate the robustness of our approach, we curated an additional dataset termed Novel Kinase Pairs (NKP). This dataset focuses on kinase-substrate combinations that are absent from the training datasets, ensuring no prior exposure to these interactions. By including novel kinases and substrates, the NKP dataset provides a rigorous test for the model’s ability to generalize to completely unseen kinase-substrate interactions, further highlighting its zero-shot learning capabilities. The results demonstrate that our model achieves state-of-the-art (SOTA) performance by leveraging full protein-kinase sequences, outperforming other methods on few-shots and zero-shot datasets, thereby showcasing its robust generalization capabilities.

In this paper, we use the terms "substrate sequence" and "protein sequence" interchangeably, considering them synonymous.

## 2 Related work

Various *in silico* prediction methods have been developed to complement experimental screening and enhance the identification of protein kinases [5]. For example, a family of algorithms called Group-based Prediction System (GPS) [33], [36], [37], [38], [7] uses machine learning methods, including penalized logistic regression, deep neural network, and Light Gradient Boosting Machine (LightGMB), to predict kinase-specific phosphorylation sites. NetPhosK [14] predicts phosphorylation sites targeted by protein kinases in peptides (7 amino acids length) using an artificial neural network (ANN) model. Quokka [18] predicts phosphorylation sites regulated by specific kinase families using an optimized logistic regression algorithm. Kim et al. Kim [15] employed Support Vector Machines (SVMs) to predict phosphorylation sites catalyzed by four protein kinase families and groups. MusiteDeep [34] was designed to predict kinase-specific phosphorylation sites using a convolutional neural network (CNN). A recent study [16] presented a unified model to provide a general framework capable of handling five kinase families. Another unified model, Phosphormer [40], [41], employed a transformer-based techniques to predict kinase-substrate interactions, which demonstrated a feasibility of zero-shot predictions on new kinases without any known substrates. Another explicit zero-shot predictor is DeepKinZero [10], which uses a bidirectional recurrent neural network to predict kinases responsible for phosphorylating specific sites on substrate proteins. However, these methods typically use peptides (consisting of 7–15 amino acids) to represent substrates or domain-level features to represent kinases [34], [40], [7],

Autoregressive transformers are a class of machine learning models designed to predict the next value in a sequence by relying on previous values [1]. These models operate under the assumption that future values in a sequence are influenced by past values, which they leverage to make predictions. In natural language processing, autoregressive models are commonly used to generate text or predict subsequent words within a sentence, learning patterns and dependencies from the training data to produce coherent, contextually appropriate text [1], [32]. Nowadays, autoregressive models like GPT [29] have become highly regarded for their effectiveness in generating high-quality text and performing diverse language tasks. In the broader protein research field, autoregressive models have shown promise, as demonstrated by the model Prot2Token [28], a general framework leveraging autoregressive language modeling to address diverse protein functions and predictions. Prot2Token effectively integrates multiple tasks within a unified architecture, showcasing the potential of autoregressive approaches to capture complex protein relationships and enhance prediction accuracy.

## 3 Materials and methods

### 3.1 Data collection and preprocessing

We utilized the dataset from GPS 6.0 which contains 24,352 phosphorylation position sites, substrate IDs, and kinase name information. We extract full substrate sequences by mapping IDs in Uniprot database. The kinase full sequences and kinase domain information were retrieved from the kinase.com database on *Homo sapiens* species. There are 221 of 387 kinases which have the kinase domain information. To reduce redundancy and ensure a diverse dataset, we applied CD-HIT [19] with a 70 % sequence similarity threshold to cluster the full protein substrate sequences based on similarity, resulting in 3588 clusters retained. The representative selection process for each cluster follows these criteria:

1. **Inter-cluster retention**: When a kinase is associated with substrate pairs across different clusters, both pairs are retained. This approach maintains diversity across clusters, ensuring that relationships between kinases and distinct substrate groups are represented.
2. **Intra-cluster deduplication**: For substrate pairs within the same cluster linked to the same kinase, only one representative pair is retained. This approach minimizes redundancy by retaining a single kinase-substrate relationship per cluster, reducing data overlap while preserving unique kinase associations within each cluster.

After this process, 11,391 unique kinase-substrate pairs remained. The data was randomly split, with 90 % allocated for training and the remaining 10 % reserved for testing using a random seed of 1. Additionally, 10 % of the training set was randomly selected as the validation set during the training process. The statistics of the training and test sets are shown in Table 1. All positive pairs represent kinase-substrate relationships where phosphorylation occurs at serine (S), threonine (T), or tyrosine (Y) sites.

**Table 1:** Dataset statistics, including number of samples for the positive training set and positive teset set.

Dataset	Number of samples
All samples	11,391
Training set	10,344
Test set	1048

Positive pairs refer to protein-kinase interactions where phosphorylation occurs at one or more sites within the full-length substrate sequence. We provide a table of data distribution before and after filtering based on number of samples, kinase groups, kinase families and kinase types (Table 2).

**Table 2:** Number of samples in the training, test, kinase groups, families and kinase types before and after filtering.

Dataset	Before filtering	After filtering
Training sample	10,812	10,344
Test samples	2460	1048
Groups	10	10
Families	98	51
Kinase types	323	78

For negative pairs, rather than randomly selecting unrelated pairs used by other methods [16], [40], [7], we define negative pairs in a way that challenges the model to distinguish between closely related kinases and substrates, promoting more nuanced and accurate predictions. We use a K-nearest neighbor approach based on Euclidean distances between kinase embeddings. This strategy ensures that negative pairs are not only distinct but also contextually challenging, forcing the model to learn nuanced differences in protein-kinase interactions. The process of creating positive and negative pairs is as follows:

### 3.2 Process of creating negative pairs

In this study, we employed two distinct approaches to generate negative samples: hard negative pairs and random negative pairs.

**Fig. 2:**
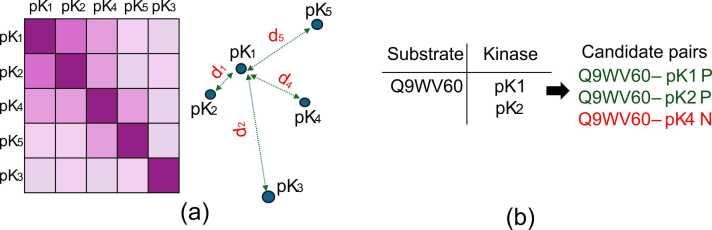
Hard negative selection process for the Q9WV60 substrate paired with kinase sequences. (a) An embedding matrix for kinases (pK1, pK2, pK3, pK4, pK5) is generated using ESM2–650M. The kinases are ranked based on Euclidean distance, creating a sorted list from closest to farthest to define the easiest to hardest negative samples. (b) In this example, the substrate Q9WV60 is paired with pK1 and pK2 as positive kinase-substrate pairs. To identify a hard negative pair, the distance matrix is used. Based on the matrix, pK4 is selected as the hardest negative sample due to its closeness to the positive pairs, making it the most challenging negative pair for effective model training.

The hard negatives approach was used for training to challenge the model with closely related kinase sequences, while randomly selected negative pairs were used for testing to ensure a diverse evaluation set. For training, our hard negative samples were designed to aid the model in learning an embedding space where Euclidean distances between kinase sequences reflect functional similarities. To enhance training efficiency, we selected challenging negative kinases by choosing those with embeddings fixed to a uniform dimension using average pooling. Negative kinases were selected based on their minimal Euclidean distance to positive kinase embeddings with phosphosites, thereby encouraging the model to learn fine-grained distinctions between positive and negative interaction. Using ESM2–650M [20] embeddings, we constructed a K-nearest neighbor matrix for each kinase, as shown in Fig. 2. For each positive pair, we selected the K-nearest negative pair from hardest value to easiest value, prompting the model to more accurately differentiate between positive and negative pairs. For each substrate, all possible kinase pairs were listed. We iterated through the list of kinase-substrate pairs for each substrate, and for each positive kinase, we selected the closest pairs as negative samples based on embedding distances.

The kinase–substrate interaction is formulated as a binary classification task, where the goal is to determine whether a connection exists between a kinase and a substrate. The training set consists of 83,013 negative samples and 10,344 positive samples, resulting in an inherently imbalanced dataset. To address this imbalance, we employed a weighted loss function, where the weight is calculated by dividing the exponentially smoothed number of negative samples by the number of positive samples.

For evaluation, negative pairs were randomly selected from kinase sequences outside the positive set of phosphosites, ensuring a broader representation of dissimilar kinases in the evaluation phase.

## 4 Model architecture

The core concept of our model is inspired by general framework of Prot2Token [28], which is designed to integrate multiple tasks within a unified architecture. The substrate-kinase interaction task can be viewed as analogous to the protein-protein interaction task in Prot2Token, with the key difference that, in this case, the model only addresses one task: substrate-kinase interaction prediction.

Our architecture consists of a bidirectional protein encoder and an autoregressive decoder, where kinase and substrate sequences are jointly processed to predict interaction labels. The substrate and kinase sequences are concatenated with an <EOS> tag between them and fed into the encoder, with a maximum combined length of 1280 amino acids. For sequences that exceed this length, both the kinase and substrate portions are proportionally trimmed. The encoder, adapted from the exact architecture ESM-2 (650 M parameters) to extract rich contextual embeddings from substrate sequences. The tokenizer operates at the character level, consisting of 33 unique tokens that represent individual amino acids along with special tokens for end-of-sequence (EOS), begin-of-sequence (BOS), masking, unknown values, and padding. The ESM-2 encoder's parameters are frozen except for the last four layers, which are fine-tuned to adapt to the kinase-substrate interaction task.

**Fig. 3:**
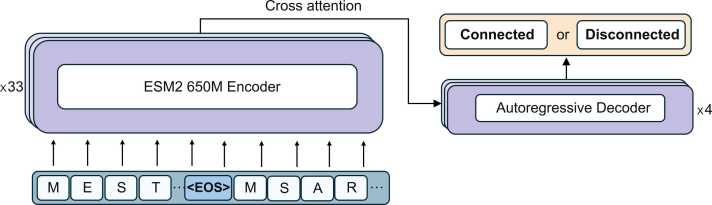
Overview of the Kinase-Substrate Interaction Model: Concatenated kinase and substrate sequences (with an <EOS> delimiter) are input into a fine-tuned, ESM-2-based encoder to extract rich contextual embeddings. These embeddings are then fused via cross-attention layers in a transformer-based decoder that autoregressively generates a binary interaction label sequence (“connected” or “disconnected”) for each kinase-substrate pair.

These embeddings are then fused with kinase and substrate sequence representations through cross-attention layers in a transformer-based decoder (640 dimensions,1280 feedforward, 8 heads, 4 layers) to model the interaction problem. The decoder receives processed embeddings from the encoder and autoregressively generates an interaction label sequence, where each token corresponds to a binary classification of *“connected”* or *“disconnected”* for the substrate-kinase pair. The model is trained, and the best checkpoints are saved until overfitting occurs. Fig. 3 shows the details of the architecture.

### 4.1 Decoder for interaction prediction

The decoder follows a causal transformer architecture with cross-attention mechanisms. Given the processed substrate-kinase representations, it sequentially predicts the interaction label as a single-token classification problem:

• **Connected (1, positive):** Indicates a biochemical interaction between the kinase and substrate.
• **Disconnected (0, negative):** Represents no interaction between the given kinase-substrate pair.

The probability of each interaction label is computed using a log-sum formulation, where the goal is to maximize the overall probability of the interaction sequence by summing the log probabilities of each predicted token. The objective function is formulated as:

(1) $$\mathrm{Maximize}\log P\left(y_{1},y_{2},\ldots ,\;y_{N}\right)=\;\sum_{t=1}^{N}\log P(y_{t}|y_{<t})$$

Where:

$P\left(y_{1},y_{2},\ldots ,\;y_{N}\right)$ represents the target label which here is “disconnected” or “connected” tokens. $P(y_{t}|y_{<t})$ indicates the conditional probability of $y_{t}$ token given the previous tokens $y_{<t}$ could be like $y_{1},y_{2},\ldots ,\;y_{t-1}$, which is all previous tokens before *t.*

The causal nature of the decoder ensures that predictions are made in an autoregressive manner, where the model progressively refines its output by attending to previously generated tokens. To optimize the model, we employ a negative log-likelihood loss function with token-level weighting to address the imbalance issue. The training objective is defined as:

(2) $$L(\theta )=-\sum_{t=1}^{T}w_{t}\log p_{\theta }\left(x_{t}|x_{1},\ldots ,x_{t-1}\right)$$

where $w_{t}$ is a learnable weighting factor that allows fine-tuning of the loss contribution for each token.

We utilize the AdamW optimizer [21] with a weight decay of 0.1, setting β₁ = 0.9, β₂ = 0.999, and an epsilon value of 1e-16 as default hyperparameters for all training sessions. The learning rate follows a cosine annealing schedule with an initial warm-up phase [21], starting at 1e-6 and gradually increasing to 5e-5 over the first 256 steps, unless otherwise specified. Training was conducted using PyTorch 2 [2] mainly on a single node equipped with four NVIDIA A100 GPUs, each with 80 GB of memory for 128 epochs with total batch size of 128 samples while other computing resources were used.

## 5 Results

In this section, we present the results and evaluation of our approach. We trained the model using both full kinase sequences and kinase domain sequences, testing various hyperparameter configurations on designated training and evaluation datasets. For both types, full kinase sequences and domain sequences, we used the same architecture and consistent hyperparameters to ensure valid comparison. Our model was evaluated on three distinct test sets: 1) an independent test set, 2) a top-k hit test set, and 3) a zero-shot test set.

### 5.1 Independent test evaluation strategy

We first evaluated our model's performance on an independent test set to assess its generalization ability to unseen data. This independent test set was carefully curated to ensure no overlap with the training dataset. The independent test set included both positive kinase-substrate pairs (with their corresponding kinase domains) and random negative pairs consisting of kinases and domains unrelated to the positive pairs.

The evaluation was conducted on a total of 2220 samples, comprising a combination of positive and negative kinase-substrate and kinase domain-substrate sequences. To comprehensively assess the model’s performance, we conducted multiple evaluations:

• **Kinase-Level Training and Evaluation** – The model was trained using full kinase sequences and evaluated on both kinase sequences and kinase domains as the input.
• **Kinase Domain-Level Training and Evaluation** – The model was retrained using kinase domain-substrate pairs as input and evaluated on the same independent test set to compare performance.

The kinase-substrate model, trained using full kinase sequences, achieved the best overall performance, with an F1-score of 0.71, accuracy of 0.72, and MCC of 0.425. This suggests that full-kinase sequence provides richer interaction features information.

We initially expected that retraining the model with kinase domains would lead to improved performance compared to models without domain-specific training. However, the experimental results showed the opposite trend. Our hypothesis is that utilizing full kinase sequences provides a more informative and comprehensive representation, capturing richer interaction features essential for accurate kinase-substrate prediction. Since the ESM-2 model was pretrained on complete sequences, it is optimized to learn a broad range of structural, functional, and interaction features across the entire protein. Using only kinase domains as input may lead to the loss of critical contextual information, including additional regulatory or binding motifs outside the core domain, resulting in a less informative representation. Retraining the model solely on kinase domains could further deviate from the original ESM-2 pretraining process, therefore leading to a decline in performance. Training with full kinase sequences achieved the best overall performance, possibly because it aligns more effectively with the model’s pretraining and leverages a more comprehensive set of features essential for accurate kinase-substrate interaction prediction.

Furthermore, to compare our model with other methods, we evaluated the PhosphormerST and GPS6 on our independent test set at the kinase level. Since GPS 6.0 is only accessible via a web portal and local installation, and PhosphormerST provides a pre-trained model with checkpoints designed solely for evaluation, we were unable to perform hyperparameter optimization for these baseline methods. We used the same dataset for all three (our model, GPS and PhosphormerST) models in the independent test evaluation strategy to ensure a fair comparison. For GPS 6.0, substrate sequences were provided as input. To incorporate kinase information during prediction, we selected each kinase group in their web server. The method generates a score for each phosphorylation site prediction, with the cutoff value varying depending on the kinase chosen. An interaction between a substrate and a kinase was considered valid if the model predicted at least one phosphorylation site in the substrate sequence. Both GPS 6.0 and PhosphormerST generate predictions at the peptide level. To consolidate these predictions at the protein level, we apply max-pooling, selecting the highest probability

On the other hand, PhosphormerST takes 15-residue peptides and kinase domains as inputs and generates a prediction score for each sample. Interactions were considered valid if the score exceeded 0.5. However, there remains a significant likelihood that our independent test set overlaps with GPS 6.0 s training data. Such overlap could artificially inflate GPS 6.0’s performance through memorization, meaning its real-world accuracy on truly novel substrate sequences may be considerably lower. Additionally, GPS 6.0 uses different cutoffs for different kinases, which may explain why it achieves lower accuracy and F1-score but a higher AUC. The results are presented in Table 3. More evaluations are presented in the Appendix.

**Table 3:** Comparison of different models for kinase-substrate interaction prediction using our independent test set.

Methods	F1-score	Accuracy	AUC	Precision	Recall	MCC
**Kinase-Substrate**	**0.71**	**0.72**	0.78	0.672	0.753	**0.425**
**Kinase domain-Substrate (not pretrain on domain)**	0.60	0.64	0.71	0.635	0.574	0.282
**Kinase domain-Substrate (train on domain)**	0.47	0.63	0.61	**0.716**	0.349	0.267
**Phosformer-ST**	0.62	0.50	0.56	0.483	0.855	0.05
**GPS 6.0**	0.64	0.62	**0.81**	0.593	0.712	0.264

Same independent test set was used to evaluate various models across F1-score, accuracy, and area under the curve (AUC). The comparison includes kinase domain-substrate models, Phosphormer (protein level), and GPS 6.0.

To provide a more direct comparison between our model and GPS 6.0, we evaluated both models using the GPS-provided test set. This dataset comprises 146 substrate-kinase pairs, each annotated with phosphorylation sites (p-sites) and kinase information. Notably, all samples in this test set belong to the *“CMGC”* kinase group. However, GPS 6.0 was able to make predictions for "p38b", "p38d", "p38g", and "p38a" kinases, which required us to exclude those samples to ensure a fair comparison. After removing these pairs, the dataset contained 100 substrate-kinase pairs in total. To maintain fairness in evaluation, we tested our model on these 100 pairs, and all reported metrics were derived accordingly. Additionally, AUC could not be computed because the dataset consists entirely of positive labels, making it impossible to assess rank-based discrimination between positive and negative samples. The results are shown in Table 4.

**Table 4:** Performance comparison with GPS6.0 on its provided test data.

Methods	F1-score	Accuracy	Recall
Our model	**0.95**	**0.91**	**0.91**
GPS6.0	0.80	0.66	0.66

### 5.2 Top-k hit analysis

In our second evaluation set, we focused on a top-k hit analysis to assess the model's performance in ranking potential kinases for each substrate sequence. For a protein substrate, where numerous potential kinases exist for each substrate, *top-k* hit provides list of possible kinases by indicating whether the model successfully ranks the true kinases among its top predictions.

The *top-k* dataset was created using a systematic approach to ensure both diversity and representativeness of the candidate kinases, through a series of preprocessing and selection steps. First, CD-HIT clustering was applied to the positive test dataset with a 40 % similarity threshold, resulting in 801 clusters. From each cluster, one representative substrate sequence was randomly selected. Next, all unique kinases from the positive training dataset were identified, totaling 387 distinct kinases. Each selected substrate sequence was paired with all 387 kinases, generating a top-k dataset containing 287,559 kinase-substrate pairs. The labels for these pairs were derived from the original positive datasets to maintain consistency with the positive data.

**Fig. 4:**
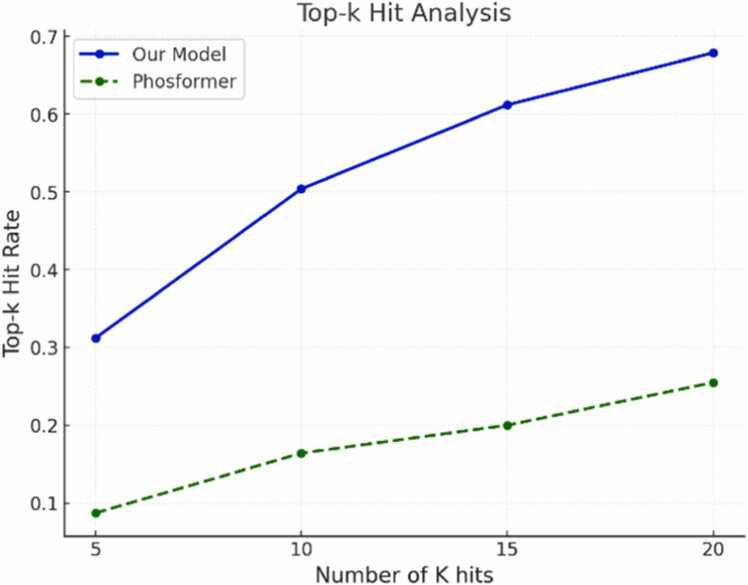
Comparison of performance between our model and Phosformer across an increasing number of K hits. Top-K hit rate indicates the probability of the true kinase among the top-K predictions.

We evaluated this *top-k* dataset using both our model and PhosformerST. Since PhosformerST provides predictions at the peptide level, we aggregated these to the protein level by selecting the maximum probability from each protein’s peptide predictions. The performance metrics for our model and Phosformer-ST across various k-values are presented in Fig. 4. Our model consistently outperforms Phosformer-ST across all k-values, with notable improvements observed as k increases. There is a significant performance gap that widens as *K* increases, demonstrating the robustness and superior ranking ability of our approach. This suggests that our model provides more accurate predictions and better generalization across kinase-substrate pairs.

### 5.3 Zero-shot evaluation

For further analysis and to evaluate the model's capabilities, we assessed its performance in a zero-shot manner using three distinct datasets designed to test its generalizability and robustness across various scenarios:

#### 5.3.1 Random split dataset

In this setup, a subset of the data was randomly withheld from the training set (using random seed 1) to serve as the test set, maintaining a 9:1 ratio of positive samples.

To incorporate negative samples into the test set, we randomly selected substrates of *Homo sapiens* phosphorylation substrates without kinase-specific phosphorylation sites from the EPSD database. This approach ensures that the test set is not included in the training set; in other words, it is an unseen subset. Additionally, the distribution of kinase groups remains consistent between the training and test sets. The negative samples for training are hard negative sets based on Euclidean distance. The number of samples in each dataset is detailed in Table 2

#### 5.3.2 Frequency-based stratified dataset

For this dataset, kinase-substrate pairs were selected based on the frequency of kinase group occurrences. Kinase groups with fewer than 1000 samples were allocated to the test set, while those with more than 1000 samples were used for training. Negative pairs were derived from the EPSD database by selecting substrates of *Homo sapiens* that contained no kinase-specific phosphorylation sites. Like the randomly split dataset, negative training samples were determined based on the Euclidean distance between kinase sequences. This configuration allows the model to be evaluated on underrepresented kinase groups, which pose a greater challenge due to limited data availability. Details are in Table 5.

**Table 5:** Statistics of zero-shot dataset.

	Training samples	Test samples	Number of groups in training	Number of groups in test
**Random Split Dataset**	9168	3076	10	10
**Frequency-based Stratified Dataset**	10,092	2616	5	5

The data consists of positive substrate-kinase pairs for training and test sets, divided based on kinase groups.

These datasets collectively provide a comprehensive framework to evaluate the zero-shot performance of our model across different scenarios, ranging from random unseen data to underrepresented and entirely novel kinase groups with a low number of samples. This multi-faceted evaluation ensures a rigorous assessment of the model's predictive capabilities.

To compare the retrained zero-shot dataset approach with our main model (general model), we have evaluated both models on a completely unseen dataset between these two models. To assess the generalization ability of the model, we curated a validation set as NKP. This dataset contains kinase-substrate pairs that were absent from both training datasets of retrained zero-shot and general model. The entire dataset contains 125 samples, including four smaller groups *(“STE”, “CK1”, “Atypical”, “TKL”)* with fewer samples, representing completely unseen novel data. This setup allows us to evaluate the model’s performance in predicting unseen interactions, simulating real-world scenarios in kinase-substrate specific prediction. The NKP dataset ensures no data leakage from the training data while enabling robust zero-shot learning evaluation. The only model that could provide zero-shot level is DeepKinZero [10]. Unfortunately, their model checkpoint is not available to test. It is important to note that our zero-shot datasets only consist of positive samples with known true labels (without known true negatives), so that AUC cannot be computed (as it requires both positive and negative samples to assess discrimination between classes). Hence, we chose F1-score, Recall, and Accuracy as the most relevant and interpretable metrics for evaluating the model’s generalization in zero-shot settings. Results for NKP dataset show the ability of the model to predict novel data and completely unseen data from training (Table 6).

**Table 6:** Zero-shot dataset evaluation results on two different zero-shot strategies.

Dataset	Recall	F1-score	Accuracy
**Random Split Dataset**	0.97	0.98	0.97
**Frequency-based Stratified Dataset**	0.75	0.86	0.75
**NKP on main model**	0.62	0.77	0.62
**NKP on frequency-based model**	0.72	0.84	0.72

## 6 Discussion

Our approach differs from traditional methods in its problem definition, focusing on protein-kinase interaction as a PPI task rather than the site-specific prediction. The superior performance of our model over existing methods can be attributed to three key innovations: (1) leveraging full kinase-substrate sequences to capture rich contextual information, (2) employing an autoregressive model for enhanced sequence representation, and (3) introducing a novel contrastive learning-inspired approach to generate challenging negative pairs. The comparison with other methods highlighted its constraints in processing capacity and kinase coverage, which our approach overcomes through a unified framework for comprehensive predictions of all kinase-substrate pairs.

The independent test results further emphasize the importance of using full kinase sequences over kinase domains. We demonstrated that kinase sequences provide richer and more informative features, contributing to improved prediction performance. This finding underscores the value of leveraging the entire sequence for tasks involving kinase-substrate interactions, as it captures both functional and evolutionary characteristics that are less apparent in kinase domain data alone. The top-k evaluation further underscores the model's effectiveness in ranking kinases for given substrates. By systematically constructing a diverse and representative top-k dataset, we demonstrated that our model consistently outperforms existing methods across various k-values. Notably, the model's ability to prioritize the true kinase among its top predictions indicates its potential utility in narrowing down candidate kinases for experimental validation.

Our zero-shot analysis where the model encounters completely unseen kinases offers further insights into the model's robustness and generalizability. By evaluating the model on datasets with varying levels of novelty, ranging from random splits to frequency-based kinase groups, we observed consistent and reliable performance across diverse scenarios. To ensure the validity of our zero-shot evaluation, we employed the NKP dataset, which was carefully curated to exclude any overlap with the training data. This rigorous approach underscores the reliability of our zero-shot scenarios and highlights the model's ability to predict novel kinase-substrate interactions effectively. We believe our method refines phosphorylation networks and supports downstream site-specific predictions by inferring novel kinase–substrate interactions with strong zero-shot performance which address real-world challenges in both basic research and therapeutic target identification[23], [35], [4], [8].

While our method allows us to leverage full substrate and kinase sequences for richer contextual information, it also presents a limitation. While larger ESM-2 representations could provide richer sequence embeddings, they require significantly more GPU memory. Additionally, the limited availability of kinase-substrate interaction datasets may constrain the performance of autoregressive models, as these models depend on data scalability to effectively learn complex interaction patterns. Another limitation is that our model was specifically developed for human, which may not be applicable to other species.

For future work, we aim to develop a more generalized model capable of addressing multiple kinase-related problems, including kinase classification, phosphorylation site prediction, and interaction filtering, to create a more comprehensive framework for kinase-substrate modeling. Additionally, we plan to incorporate structural information alongside sequence-based embeddings, as structural features play a critical role in kinase specificity and function. By integrating sequence and structural data, we aim to enhance model accuracy and generalization across diverse kinase families and species.

## CRediT authorship contribution statement

**Esmaili Farzaneh:** Writing – review & editing, Writing – original draft, Validation, Software, Methodology, Investigation, Formal analysis, Data curation, Conceptualization. **Xu Dong:** Writing – review & editing, Supervision, Project administration, Investigation, Funding acquisition, Conceptualization. **Wang Duolin:** Writing – review & editing, Validation, Software, Methodology, Investigation, Formal analysis, Data curation. **Qin Yongfang:** Validation, Software.

## Author contributions

**F.E.** developed algorithms, and wrote manuscript. **Y.Q.** evaluated different tools. **D.W.** provided technical input and revised the manuscript. **D.X.** conceived and supervised the study, and revised the manuscript. All authors have read and agreed to the published version of the manuscript.

## Declaration of Competing Interest

The authors declare that they have no conflict of interest.

## Figures and Tables

**Table 7:** Comparison between hard negative vs random negative on training data. The results are evaluated on the validation set

	Random negative		Hard Negative	
	F1-score	Accuracy	F1-score	Accuracy
Our model	0.54	37.33	0.86	75.45

**Fig. 5:**
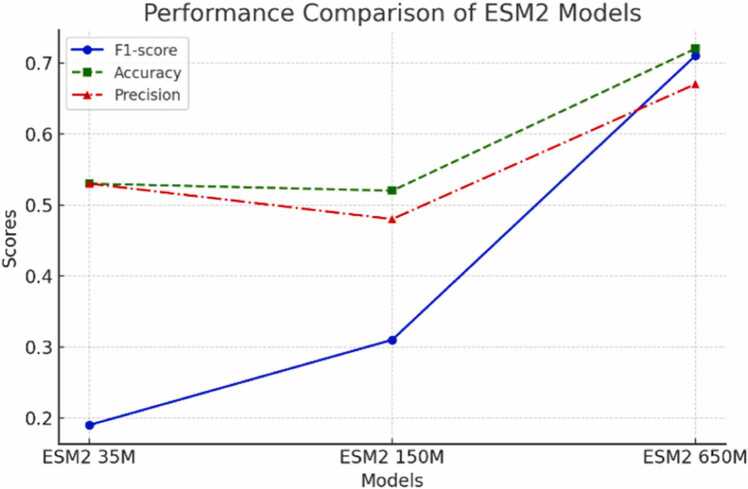
Performance comparison among different ESM2 models on our independent test set

**Fig. 6:**
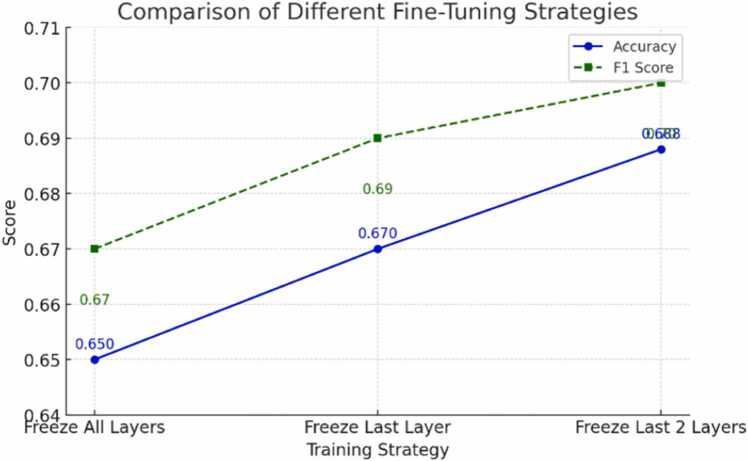
To compare different decoder layers, we have different experiments: 1. Use freezing ESM2 650 M, 2. Freeze all layers except the last layer, 3. Freeze all layers except 2 layers

## Appendix

### Ablation Study

To assess the impact of negative sample selection on model performance, we compared training with random negative pairs versus hard negative pairs and evaluated the model on validation set. Results are shown in Table 7.

Additionally, we experimented with multiple ESM-2 model sizes—ESM2–35M, ESM2–150M, and ESM2–650M—using our training data and evaluating on an independent test set with hard negative training samples, in order to analyze the trade-off between model complexity, computational efficiency, and predictive performance; note that the architecture of all ESM-2 models is identical to the main model trained with 650 M parameters. Fig. 5 shows the result.

To compare different decoder layers, we have different experiments: 1. Use freezing ESM-2 650 M, 2. Freez all layers except the last layer, 3. Freez all layers except 2 layers. The training, validation and test set are similar the main model. The results show in Fig. 6.
